# Respiratory muscle work influences locomotor convective and diffusive oxygen transport in human heart failure during exercise

**DOI:** 10.14814/phy2.14484

**Published:** 2020-06-19

**Authors:** Joshua R. Smith, Jessica D. Berg, Timothy B. Curry, Michael J. Joyner, Thomas P. Olson

**Affiliations:** ^1^ Department of Cardiovascular Medicine Mayo Clinic Rochester MN USA; ^2^ Department of Anesthesiology and Perioperative Medicine Mayo Clinic Rochester MN USA

**Keywords:** leg blood flow, oxygen transport, respiratory muscle metaboreflex, work of breathing

## Abstract

**Introduction:**

It remains unclear if naturally occurring respiratory muscle (RM) work influences leg diffusive O_2_ transport during exercise in heart failure patients with reduced ejection fraction (HFrEF). In this retrospective study, we hypothesized that RM unloading during submaximal exercise will lead to increases in locomotor muscle O_2_ diffusion capacity (D_M_O_2_) contributing to the greater leg VO_2_.

**Methods:**

Ten HFrEF patients and 10 healthy control matched participants performed two submaximal exercise bouts (i.e., with and without RM unloading). During exercise, leg blood flow was measured via constant infusion thermodilution. Intrathoracic pressure was measured via esophageal balloon. Radial arterial and femoral venous blood gases were measured and used to calculate leg arterial and venous content (CaO_2_ and CvO_2_, respectively), VO_2_, O_2_ delivery, and D_M_O_2_.

**Results:**

From CTL to RM unloading, leg VO_2_, O_2_ delivery, and D_M_O_2_ were not different in healthy participants during submaximal exercise (all, *p* > .15). In HFrEF, leg VO_2_ (CTL: 0.7 ± 0.3 vs. RM unloading: 1.0 ± 0.4 L/min, *p* < .01), leg O_2_ delivery (CTL: 0.9 ± 0.4 vs. RM unloading: 1.4 ± 0.5 L/min, *p* < .01), and leg D_M_O_2_ (CTL: 31.5 ± 11.4 vs. RM unloading: 49.7 ± 18.6 ml min^−1^ mmHg^−1^) increased from CTL to RM unloading during submaximal exercise (all, *p* < .01), whereas CaO_2_‐CvO_2_ was not different (*p* = .51). The degree of RM unloading (i.e., % decrease in esophageal pressure‐time integral during inspiration) was related to the % increase in leg D_M_O_2_ with RM unloading (*r* = −.76, *p* = .01).

**Conclusion:**

Our data suggest RM unloading leads to increased leg VO_2_ due to greater convective and diffusive O_2_ transport during submaximal exercise in HFrEF patients.

## Graphical abstract

Respiratory muscle work negatively influences convective O_2_ transport in heart failure patients. The present study investigated the impact of respiratory muscle work on leg O_2_ diffusive capacity during submaximal exercise. We found the degree of respiratory muscle unloading in heart failure patients is related to the improvement in leg O_2_ diffusive capacity during submaximal exercise.

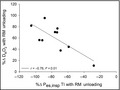

## 1 INTRODUCTION

Heart failure patients with reduced ejection fraction (HFrEF) exhibit impaired exercise tolerance, which is a hallmark symptom of patients with HFrEF. The underlying pathophysiologic mechanisms responsible for this compromised exercise tolerance are multifactorial, but include impaired cardiac output and exaggerated sympathetically mediated vasoconstriction in the periphery, which limits locomotor muscle blood flow (Q_L_; Poole, Hirai, Copp, & Musch, 2012). In addition, patients with HFrEF often present with pulmonary abnormalities including obstructive‐restrictive lung disorders, lower lung diffusion capacity, increased physiologic dead space, and ventilation/perfusion mismatch (Olson, Snyder, & Johnson, 2006; Poole et al., 2012; Smith & Olson, 2019). Importantly, HFrEF patients, compared to healthy adults, have an augmented ventilatory response (i.e., ↑ ventilatory equivalent for carbon dioxide slope), respiratory muscle work, and subsequent cardiac output distribution to the respiratory muscles (i.e., diaphragm) during exercise (Agostoni, Cattadori, Bianchi, & Wasserman, 2003; Cross, Sabapathy, Beck, Morris, & Johnson, 2012; Musch, 1993; Olson et al., 2010; Smith, Hageman, Harms, Poole, & Musch, 2017; Smith et al., 2020).

The high respiratory muscle work and blood flow coupled with the limited cardiac output reserve during exercise in these patients significantly impacts cardiac output distribution and consequently contributes to exercise intolerance (Borghi‐Silva et al., 2008; Musch, 1993; O'Donnell, D'Arsigny, Raj, Abdollah, & Webb, 1999; Olson et al., 2010; Smith et al., 2017). Specifically, Olson and colleagues found that unloading the naturally occurring respiratory muscle work during submaximal exercise in patients with HFrEF resulted in greater leg oxygen uptake (VO_2_). This respiratory muscle unloading‐induced increase in leg VO_2_ was due to greater Q_L_, total cardiac output (Q_T_), and Q_L_ as a percent of Q_T_ (%Q_L_), as leg arteriovenous oxygen difference (CaO_2_‐CvO_2_) was not different compared to control (i.e., without respiratory muscle unloading; Olson et al., 2010). As such, these findings suggest that the respiratory muscle unloading‐induced increase in convective O_2_ transport was primarily responsible for these findings. However, HFrEF patients exhibit exaggerated sympathetically mediated vasoconstriction coupled with microvascular abnormalities that compromise diffusive O_2_ transport (Behnke, Delp, Poole, & Musch, 2007; Esposito, Mathieu‐Costello, Shabetai, Wagner, & Richardson, 2010; Poole, Copp, Hirai, & Musch, 2011; Poole et al., 2012; Richardson, Kindig, Musch, & Poole, 2003). Specifically, HFrEF is associated with impaired capillary hemodynamics at rest and during exercise constraining muscle O_2_ diffusing capacity (D_M_O_2_). Importantly, superfusion of sodium nitroprusside prior to electrically stimulated muscle contractions in HFrEF rats increased microvascular PO_2_ (i.e., driving pressure for O_2_ from blood to myocyte) during contractions to a similar level reported in the sham rats (Ferreira et al., 2006) suggesting that increases in Q_L_ can improve the microvascular abnormalities in HFrEF. As high respiratory muscle work contributes to sympathetically mediated vasoconstriction in HFrEF (Chiappa et al., 2008; Olson et al., 2010), it is plausible that partial alleviation of the sympathetically mediated vasoconstriction via respiratory muscle unloading may also improve D_M_O_2_. These proposed findings would have important clinical implications as they would suggest that interventions to “unload” the respiratory muscles (e.g., inspiratory muscle training) would improve both convective and diffusive O_2_ transport in patients with HFrEF.

As mentioned earlier, Olson et al. determined the influence of respiratory muscle unloading on convective O_2_ transport during submaximal exercise in patients with HFrEF; however, D_M_O_2_ was not investigated. Therefore, the purpose of this study was to retrospectively examine data from this previous study (Olson et al., 2010) and specifically quantify the influence of respiratory muscle work on D_M_O_2_ during submaximal exercise in patients with HFrEF. We hypothesized that respiratory muscle unloading during submaximal exercise would result in greater D_M_O_2_ compared to control in HFrEF patients. Further, we hypothesized that the degree of respiratory muscle unloading would be significantly related to the improvement in D_M_O_2_ during exercise.

## 2 METHODS

### 2.1 Participants

Ten HFrEF patients were recruited from the Mayo Clinic Heart Failure Service and the Cardiovascular Health Clinic and 10 healthy matched adult participants were recruited as previously described (Olson et al., 2010). Briefly, inclusion criteria for the HFrEF patients included diagnosis of ischemia or dilated cardiomyopathy with duration of >1 year of symptoms, stable HF symptoms (>3 months), left ventricular ejection fraction ≤35%, body mass index of <35 kg/m^2^, nonsmokers with a smoking history of <15 pack‐years, and no diagnosis of coexisting pulmonary disease or taking medications. All aspects of this study were approved by the Mayo Clinic Institutional Review Board and conformed to the standards set forth by the latest revision of the Declaration of Helsinki. All participants were informed about the experimental procedures and potential risk involved, and provided written and verbal informed consent.

### 2.2 Experimental design

As previously described (Olson et al., 2010), participants performed all protocols and measurements during two study visits. On the first study visit, participants were first familiarized with all experimental measurements and protocols and then completed an incremental cycle ergometry exercise test to volitional fatigue to determine peak oxygen uptake (VO_2_peak). On the second study visit, participants performed two steady‐state exercise sessions at 60% peak workload. The first exercise session consisted of 3 min of rest followed by 15 min of constant load cycling. During the first and third 5 min, the participants breathed normally under room air conditions. During the second 5 min, the respiratory muscles were unloaded with the assistance of a mechanical ventilator during inspiration. The first and second exercise session protocols were similar except the second 5 min of the second exercise session consisted of respiratory muscle loading via inspiratory resistance. The primary focus of this study was to determine if respiratory muscle *unloading* influences leg D_M_O_2_ during exercise in HFrEF patients. As such, only the data from the exercise session with respiratory muscle unloading are reported herein.

As previously described in depth (Olson et al., 2010), Q_L_ was measured via constant infusion thermodilution, intrathoracic pressure via esophageal balloon, arterial blood pressure via radial arterial catheter, arterial and femoral venous blood gases via radial arterial and femoral venous blood sampling, and Q_T_ via open‐circuit acetylene wash‐in technique.

### 2.3 Calculated variables

Radial arterial and femoral venous blood sampling occurred anaerobically over 10–15 s during control and unloading exercise for measurements of partial pressure of oxygen (PaO_2_ and PvO_2_), hemoglobin (Hb), and saturation of oxygen (SaO_2_ and SvO_2_; IL‐1620, Instrumentation Laboratories). Blood gases were analyzed in duplicate, averaged, and temperature corrected at a temperature of 37°C. Direct measures assessed via blood sampling were used to calculate leg arterial and venous content [CaO_2_ = (1.34 × Hb × SaO_2_) + (PaO_2_ × 0.0031) and CvO_2_ = (1.34 × Hb × SvO_2_) + (PvO_2_ × 0.0031)]. Leg VO_2_ was calculated as Q_L_ multiplied by leg CaO_2_‐CvO_2_. Leg O_2_ delivery was calculated as Q_L_ multiplied by CaO_2_. Leg O_2_ diffusion capacity (D_M_O_2_) was calculated via Fick's Law of Diffusion, VO_2_ = D_M_O_2_ × (P_cap_O_2_ − P_mit_O_2_), where P_cap_O_2_ and P_mit_O_2_ are mean capillary and mitochondrial PO_2_, respectively. During submaximal exercise (~50%–60% VO_2_peak), previous studies have found that P_cap_O_2_ is proportional to PvO_2_ and P_mit_O_2_ is ~1–3 mmHg (and thus was assumed to be zero; Honig, Gayeski, Clark, & Clark, 1991; Richardson, Noyszewski, Kendrick, & Leigh, 1995; Roca et al., 1985). As such, Fick's Law of Diffusion was simplified as VO_2_ = DO_2_ × PvO_2_ (Ade, Broxterman, Moore, & Barstow, 2017; Esposito et al., 2010). It should be noted that the previous studies examining myoglobin PO_2_ during exercise were conducted in healthy adults or animal models. It was assumed in this study that similar myoglobin PO_2_ levels are reached during submaximal exercise in HFrEF. Furthermore, we recognize that the simplification of Fick's Law of Diffusion and use of PvO_2_ will lead to higher D_M_O_2_ values compared to when P_cap_O_2_ is used because P_cap_O_2_ is systematically higher than PvO_2_ (Roca et al., 1985).

### 2.4 Statistical analyses

Values are reported as mean ± standard deviation (*SD*). Statistical analyses were performed using SigmaStat 2.0 (Jandel Scientific). Normality and equal variance were assessed using the Shapiro–Wilk and Levene tests, respectively, and nonparametric tests were used when appropriate. Cardiovascular variables were compared within (control vs. respiratory muscle unloading) and between groups (HFrEF vs. healthy participants) using mixed factorial analysis of variance and Tukey's post hoc test when appropriate. Relationships were determined via linear regression. Statistical significance was set at *p* < .05.

## 3 RESULTS

### 3.1 Participant characteristics

As previously described (Olson et al., 2010), the patients with HFrEF had a mean age of 54 ± 15 years, left ventricular ejection fraction of 31 ± 8%, VO_2_peak of 17 ± 5 ml kg^−1^ min^−1^ and ischemic (*n* = 5) and idiopathic (*n* = 5) etiologies. Medications for the HFrEF patients included angiotensin converting enzyme inhibitors (*n* = 5), beta blockers (*n* = 9), digitalis (*n* = 3), aspirin (*n* = 7), and diuretics (*n* = 6). Lastly, age, height, weight, and sex were not different between the healthy participants and HFrEF patients (Olson et al., 2010).

### 3.2 Leg O_2_ transport with RM unloading

**FIGURE 1:**
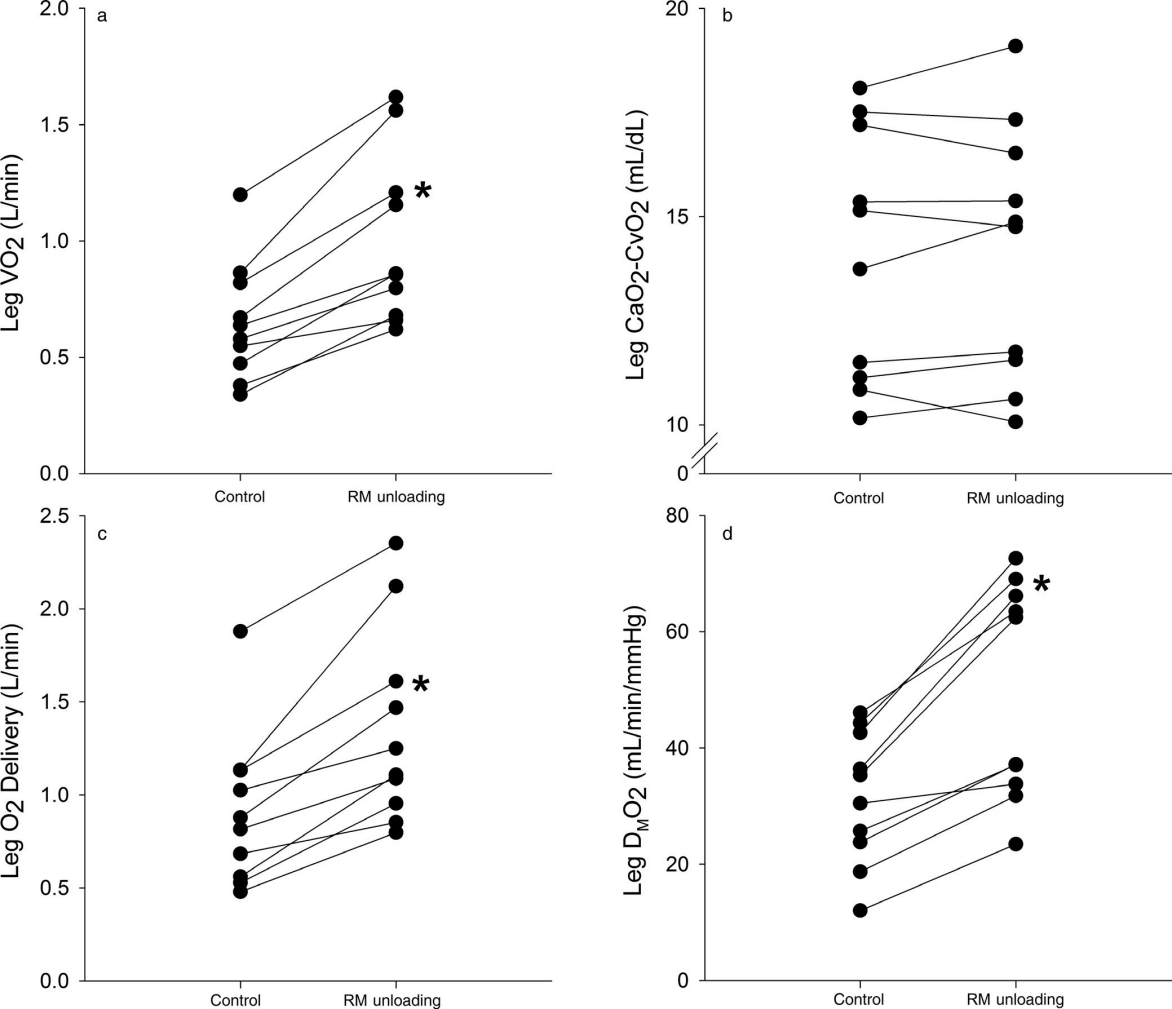
O_2_ delivery and utilization in heart failure patients with reduced ejection fraction during submaximal exercise. The individual responses with control and respiratory muscle (RM) unloading for leg VO_2_ (a), CaO_2_‐CvO_2_ (b), O_2_ delivery (c), and D_M_O_2_ (d). Leg VO_2_, O_2_ delivery, and D_M_O_2_ increased from control to RM unloading (all, *p* < .01), whereas CaO_2_‐CvO_2_ was not different (*p* = .51). * significantly different from control

**FIGURE 2:**
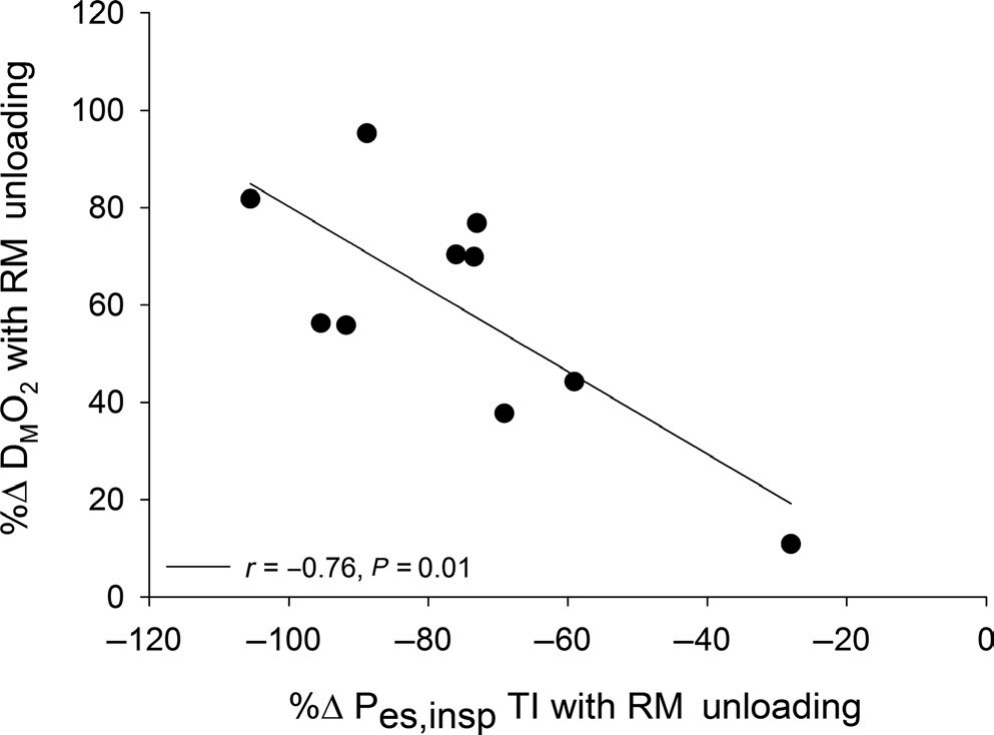
Relationship between intrathoracic pressure and leg D_M_O_2_ with respiratory muscle unloading. There was a negative relationship between the %Δ in inspiratory esophageal pressure time integral (P_es,insp_TI) and %Δ in leg D_M_O_2_ with respiratory muscle (RM) unloading compared to control (*r* = −.76, *p* = .01)

For the healthy participants, leg VO_2_ (control: 1.5 ± 0.8 versus. RM unloading 1.5 ± 0.9 L/min), O_2_ delivery (control: 2.1 ± 1.2 vs. RM unloading 2.1 ± 1.2 L/min), and D_M_O_2_ (control: 64.5 ± 37.4 vs. RM unloading 68.5 ± 41.4 ml min^−1^ mmHg^−1^) were not different between control and respiratory muscle unloading (all, *p* > .15), whereas CaO_2_‐CvO_2_ was higher with respiratory muscle unloading (control: 14.0 ± 1.0 vs. RM unloading 14.4 ± 1.2 ml/dl; *p* = .03). Figure 1 shows leg VO_2_, CaO_2_‐CvO_2_, O_2_ delivery, and D_M_O_2_ during submaximal exercise with control and respiratory muscle unloading in HFrEF patients. With respiratory muscle unloading, leg VO_2_, O_2_ delivery, and D_M_O_2_ increased in all HFrEF patients by ~55%–60% compared to control (all, *p* < .01). Furthermore, the % change in P_es,insp_ TI with respiratory muscle unloading was negatively related to the % change in D_M_O_2_ with respiratory muscle unloading (compared to control) during submaximal exercise (*r* = −.76, *p* = .01; Figure 2). In contrast, leg CaO_2_‐CvO_2_ was not different with respiratory muscle unloading compared to control (*p* = .51).

## 4 DISCUSSION

### 4.1 Major findings

The purpose of this retrospective study was to determine if partially unloading the naturally occurring respiratory muscle work influenced locomotor muscle O_2_ diffusing capacity (D_M_O_2_) in addition to convective O_2_ transport during submaximal exercise in HFrEF patients. The principal novel finding of this study was that respiratory muscle unloading resulted in greater D_M_O_2_ during submaximal exercise in HFrEF, but not healthy participants. Second, the increase in D_M_O_2_ was significantly related to the degree of respiratory muscle unloading during submaximal exercise in the patients with HFrEF. These results indicate that the naturally occurring respiratory muscle work in HFrEF patients contributes to impaired leg VO_2_ during submaximal exercise via impairments in convective *and* diffusive O_2_ transport. Furthermore, these findings have important clinical implications as they suggest that interventions (e.g., inspiratory muscle training) aimed at ameliorating the respiratory muscle metaboreflex‐induced consequences on leg convective O_2_ transport will likely also improve diffusive O_2_ transport.

### 4.2 Respiratory muscle work and diffusive O_2_ transport

**FIGURE 3:**
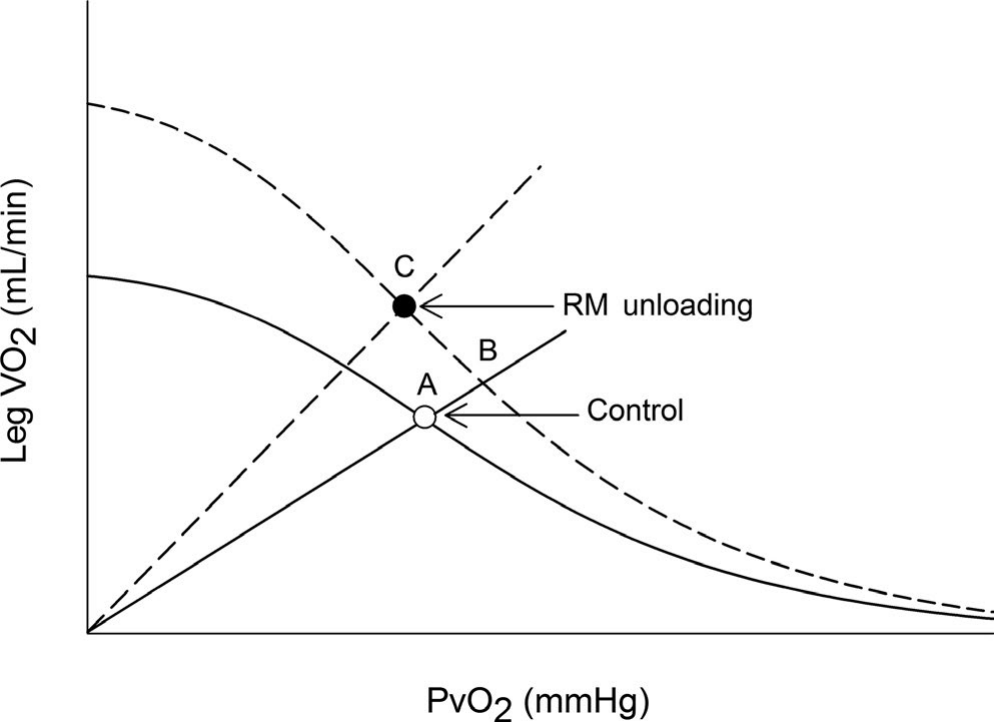
Illustration of the convective and diffusive components that integrate to determine VO_2_ with control and respiratory muscle unloading. This model integrates convective O_2_ (Fick Principal, curved lines) and diffusive O_2_ components (Fick's Law, straight lines from origin) to determine leg VO_2_ for control and respiratory muscle (RM) unloading. With RM unloading, the curved Fick Principle line is higher because of the increases in convective O_2_ delivery. Furthermore, there was greater leg diffusing O_2_ capacity (D_M_O_2_) with RM unloading compared to control (i.e., the slope of the straight Fick's Law line was greater with RM unloading than control). If RM unloading increased leg VO_2_ during submaximal exercise in HFrEF solely due to greater convective O_2_ delivery than leg VO_2_ would have moved from A to B. However, the greater D_M_O_2_ with RM unloading presented herein suggests that the RM unloading‐induced increase in leg VO_2_ was due to both greater convective and diffusive O_2_ transport (A to C)

In this study, we found that unloading the naturally occurring respiratory muscle work increased D_M_O_2_ by ~60% during submaximal exercise in HFrEF patients. Furthermore, we found that the increase in D_M_O_2_ was associated with the degree of respiratory muscle unloading. These data in concert with those showing that respiratory muscle unloading leads to increases in Q_T_, Q_L_, and %Q_L_ (Olson et al., 2010) suggest the HFrEF‐induced respiratory muscle work during submaximal exercise impairs leg VO_2_ by altering both convective and diffusive O_2_ transport. Figure 3 illustrates the integration of convective and diffusive O_2_ transport in determining leg VO_2_ during submaximal exercise. As previously described (Ade et al., 2017; Poole et al., 2012; Wagner, 1991, 1996), the curve line represents convective O_2_ transport described with Fick Principal and the straight line represents D_M_O_2_ described with Fick's Law of Diffusion with the intersecting point representing leg VO_2_. If unloading the respiratory muscles increased leg VO_2_ only via increases in convective O_2_ transport, leg VO_2_ would have increased from A to B. However, respiratory muscle unloading also increased D_M_O_2_ revealing that the combined increases in convective and diffusive O_2_ transport led to greater increases in leg VO_2_ (from A to C). In contrast to D_M_O_2_, respiratory muscle unloading did not alter leg Ca‐CvO_2_ during submaximal exercise. As O_2_ utilization = 1 − $e^{\;-D_{M}O_{2}/\left(xQ_{L}\right)}$ (with β as the linear approximation to the slope of the O_2_ dissociation curve; Poole et al., 2012; Wagner, 1991), changes in both Q_L_ and D_M_O_2_ influence changes in leg Ca‐CvO_2_. Because Q_L_ and D_M_O_2_ increased to a similar extent (and thus the ratio between them was similar), it is then not surprising that leg Ca‐CvO_2_ was not different between conditions.

The primary factors that determine diffusion of O_2_ across a membrane (as indicated in Fick's Law of Diffusion) include (a) physical properties of the gas, (b) membrane thickness, (c) surface area available for diffusion, and (d) the partial pressure gradient across the membrane with the latter two likely contributing to the findings presented herein. Specifically, Richardson et al. found that HFrEF rats exhibited a reduction in the percentage of capillaries that support red blood cells at rest and during electrically elicited contractions compared to control rats (Richardson et al., 2003). Furthermore, the HFrEF rats had blunted increases in capillary red blood cell flux and velocity compared to control rats with contractions (Richardson et al., 2003). Importantly, the lower capillary red blood cell velocity in HFrEF compared to control rats will reduce surface area available for capillary‐myocyte O_2_ exchange and thereby D_M_O_2_ (Poole et al., 2011). The pathophysiological mechanisms responsible for this impaired capillary hyperemic response in HFrEF are multifactorial with exaggerated sympathetically mediated vasoconstriction having a contributory role (Richardson et al., 2003). This disruption in capillary hemodynamics in HFrEF rats subsequently results in lower microvascular PO_2_ (i.e., driving pressure for O_2_ from blood to myocyte) during muscle contractions (Behnke et al., 2007; Ferreira et al., 2006). Crucially, superfusion of sodium nitroprusside prior to electrically stimulated muscle contractions increased microvascular PO_2_ at baseline and during contractions in the HFrEF rats to a similar level reported in the sham rats (Ferreira et al., 2006) suggesting that increases in muscle blood flow can ameliorate the pathophysiologic microvascular PO_2_ response in HFrEF. As previously described (Olson et al., 2010), unloading the respiratory muscles in HFrEF patients during submaximal exercise resulted in decreased leg vascular resistance facilitating greater Q_L_ and O_2_ transport. Taken together, attenuating the exaggerated sympathetically mediated vasoconstriction via respiratory muscle unloading likely increased red blood cell velocity improving both surface area for capillary‐myocyte O_2_ exchange and microvascular PO_2_ during submaximal exercise; however, future studies are necessary to confirm this hypothesis in human HF.

The improvements in convective and diffusive O_2_ transport with respiratory muscle unloading culminating in greater leg VO_2_ during submaximal exercise in patients with HFrEF have important clinical significant implications. Consistent with previous studies (Sullivan, Knight, Higginbotham, & Cobb, 1989; Zelis, Longhurst, Capone, & Mason, 1974), these findings indicate that leg VO_2_ is impaired during submaximal exercise in patients with HFrEF resulting in a greater reliance on anaerobic metabolism thereby precipitating fatigue development. In this study, the % change in leg VO_2_ was negatively related to the % change in blood pH with respiratory muscle unloading (compared to control; *r* = −.75, *p* = .02) suggesting the respiratory muscle unloading‐induced higher leg VO_2_ was related to higher oxidative metabolism thereby sparring anaerobic energy sources. These findings highlight the potential impact of interventions focused on improving respiratory muscle function (e.g., inspiratory muscle training) may have on exercise tolerance and exertional symptomology for patients with HFrEF.

## 5 CONCLUSIONS

During submaximal exercise, respiratory muscle unloading resulted in greater D_M_O_2_ HFrEF patients. On the basis of these data, we conclude that the respiratory muscle unloading‐induced increases in leg VO_2_ is due to greater convective *and* diffusive O_2_ transport. Future prospective studies are necessary to determine if clinically pertinent interventions aimed at unloading the respiratory muscles (e.g., inspiratory muscle training) also improves diffusive O_2_ transport in HFrEF patients.

## CONFLICT OF INTEREST

There are no conflicts of interest to report.

## AUTHOR CONTRIBUTIONS

JRS, TBC, MJJ, and TPO conceived and designed the research. TBC, MJJ, and TPO performed the experiments. JRS and JDB analyzed the data, interpreted the results of the experiment, and prepared figures. JRS drafted the manuscript. All authors edited, revised, and approved the final version of the manuscript.
